# Validation of weak biological effects by round robin experiments: cytotoxicity/biocompatibility of SiO_2_ and polymer nanoparticles in HepG2 cells

**DOI:** 10.1038/s41598-017-02958-9

**Published:** 2017-06-28

**Authors:** Lisa Landgraf, Daniel Nordmeyer, Peter Schmiel, Qi Gao, Sandra Ritz, Julia S. Gebauer, Stefan Graß, Silvia Diabaté, Lennart Treuel, Christina Graf, Eckart Rühl, Katharina Landfester, Volker Mailänder, Carsten Weiss, Reinhard Zellner, Ingrid Hilger

**Affiliations:** 1 Department of Experimental Radiology, Institute of Diagnostic and Interventional Radiology I, University Hospital Jena, Friedrich-Schiller Universität Jena, Am Klinikum 1, 07747 Jena, Germany; 2 Physikalische Chemie, Hochschule Darmstadt, University of Applied Sciences, Fachbereich Chemie und Biotechnologie, Hochschulstrasse 2, 64289 Darmstadt, Germany; 3 Max Planck Institute for Molecular Biology, Ackermannweg 10, 55128 Mainz, Germany; 4 Institute of Physical Chemistry, University of Duisburg-Essen, 45128 Essen, Germany; 5 Department of Dermatology, University Medicine of the Johannes-Gutenberg University Mainz, Langenbeckstr. 1, 55131 Mainz, Germany; 6 Karlsruhe Institute of Technology, Institute of Toxicology and Genetics, Hermann-von-Helmholtz-Platz 1, 76344 Eggenstein-Leopoldshafen, Germany; 7 Fraunhofer ICT-IMM, Carl-Zeiss-Str. 18-20, 55129 Mainz, Germany

## Abstract

All over the world, different types of nanomaterials with a diversified spectrum of applications are designed and developed, especially in the field of nanomedicine. The great variety of nanoparticles (NPs), *in vitro* test systems and cell lines led to a vast amount of publications with conflicting data. To identify the decisive principles of these variabilities, we conducted an intercomparison study of collaborating laboratories within the German DFG Priority Program SPP1313, using well-defined experimental parameters and well-characterized NPs. The participants analyzed the *in vitro* biocompatibility of silica and polymer NPs on human hepatoma HepG2 cells. Nanoparticle mediated effects on cell metabolism, internalization, and inflammation were measured. All laboratories showed that both nanoparticle formulations were internalized and had a low cytotoxicity profile. Interestingly, small variations in nanoparticle preparation, cell handling and the type of culture slide influenced the nanoparticle stability and the outcomes of cell assays. The round robin test demonstrated the importance of the use of clearly defined and characterized NPs and parameters for reproducible results across laboratories. Comparative analyses of *in vitro* screening methods performed in multiple laboratories are absolutely essential to establish robust standard operation procedure as a prerequisite for sound hazard assessment of nanomaterials.

## Introduction

The ubiquity of NPs in daily life as well as their utilization as versatile tools in biomedicine has received increased attention among scientists in the field of nanotechnology. In this context, particularly non-metal oxide NPs, such as amorphous silica (SiO_2_) and polymeric NPs are considered for a broad range of applications, such as additives to drugs, cosmetics, varnishes, and food, or as tools for DNA delivery^1^, the identification of biomarkers^2^, and drug delivery^3^.

As true for many nanomaterials silica NPs often aggregate in cell culture media. This depends not only on the surface functionalization but also on the transfer protocol from the initial synthesis/storage medium (usually water or ethanol) or the dried state to the cell culture medium^4^. In many studies, the aggregation state in cell culture medium is not controlled at all and hence, impedes a comparison of different cell culture studies.

The advances in polymer science allow producing well-defined nanoparticle formulations in a wide range concerning size, shape, and sub-particle morphology^5, 6^. For polymer NPs polyesters such as polylactides, poly(ε-caprolacton), polyglycolides and their copolymers are widely used as long-circulating drug carriers due to their biodegradability and low toxicity *in vivo* ^7–10^. Polystyrene, because of its low cost and availability, is mainly used for *in vitro* cell uptake experiments as a model^11, 12^. Complex NPs or nanocapsules are thought to be ideally suited for applications in biomedical application as marker and targeted drug-delivery system^13^. The great variety of parameters and the lack of standardized protocols often preclude a comprehensive understanding of the factors governing the biological impact of nanoparticles in cells *in vitro*. In view of the different experimental conditions and read-outs under which cytotoxicological investigations of NPs have been performed it is not surprising that the results related to the biological impact of these NPs on cells are rather inconsistent and hence inconclusive. Such inconsistencies prevent the elucidation of fundamental and molecular rules which govern the potential pathophysiological responses to NP exposure in living organisms.

A major obstacle for the safe development and exploitation of nanotechnology is the lack of standardized procedures to investigate potential adverse effects of nanomaterials. As demonstrated for the two selected examples, silica and polymeric NPs, hazard assessment remains inconclusive due to differences in nanomaterial properties, cell types or lines, read-outs, and preparation of nanoparticle suspensions. Furthermore, it is already known that although different laboratories may have used the same assay and nanomaterial for testing in the same cell line, variations in the experimental procedures still lead to inconsistent results. In view of this situation we have decided to perform a carefully controlled round-robin experiment involving a number of different laboratories in Germany. In this intercomparison experiment we studied quasi-monodisperse NPs with a diameter in a similar size range (TEM diameter: 55 ± 2 nm and 74 ± 11 nm, hydrodynamic diameter about 120 nm) but of different chemical composition i.e. silica and polystyrene NPs, two of the most frequently used nanomaterials. The particles used were synthesized and characterized in our laboratories using both transmission electron microscopy (TEM) and dynamic light scattering (DLS). As a cell model we choose the human HepG2 cell line, since the liver is one of the most critical targets of NPs, especially in the field of nanomedicine. As read-outs for cell viability we decided to include the most widely used assays to monitor i) cellular dehydrogenase activity and ATP-levels as well as ii) LDH release. In addition, we monitored the release of interleukin-6 (IL-6) and interleukin-8 (IL-8) as markers of inflammation. To measure nanoparticle uptake in cells confocal laser scanning microscopy and flow cytometry were employed. The collaborating laboratories involved in this intercomparison study were all part of the German DFG Priority Program SPP1313 “Biological Responses to Nanoscale Particles”^14^ and involved laboratories at

- The Freie Universität Berlin (Lab 1)
- The University Clinic of Mainz and the Max-Planck-Institute of Mainz (Lab 2)
- The University of Jena (Lab 3)
- The University of Duisburg-Essen (Lab 4)
- The Karlsruhe Institute of Technology (Lab 5).

## Materials and Methods

### Chemicals

**A**ll chemicals used in this synthesis of SiO_2_ NPs were applied as received without further purification: hydrofluoric acid (Fluka, 40–45%), ammonia solution (Carl Roth, *puriss*., 25%), 3-(aminopropyl)trimethoxysilane (APS, Sigma-Aldrich, 97%), ethanol (Berkel AHK, *puriss*., 100%), fluorescein isothiocyanate (FITC, Fluka, 99%), Igepal CO-520 (Sigma-Aldrich, 99%), cyclohexane (Carl Roth p.a., ≥99.5%), and tetraethyl orthosilicate (TEOS, Sigma-Aldrich, ≥99.0%). Styrene (99%, Merck, Germany) and acrylic acid (AA, 99%, Aldrich) were freshly distilled under reduced pressure and stored at −18 °C before use. Other reagents and solvents were commercial products and were used without further purification: n-hexadecane, HD (Sigma-Aldrich), initiator 2,2′-azobis(2-methylbutyronitrile), V59 (Wako Chemicals, Germany), sodium dodecyl sulfate, SDS (99%, Sigma-Aldrich). Ultrapure water with a measured resistance of 18.2 MΩ∙cm was obtained from a Millipore purification system (Milli-Q^®^ Academic). Dulbecco’s Modified Eagle Medium (DMEM) with 10% fetal calf serum (FCS) (Life Technologies) was used from the consortium stockpile as described above. Standard cell culture media (Dulbecco’s Modified Eagle Medium (DMEM) with 10% fetal calf serum (FCS) were purchased from Invitrogen. The cell culture medium and the HepG2 cells (CLS- Cell Lines Service, Germany) were purchased by Lab 3 and distributed to all groups in the consortium.

### Synthesis and characterization of SiO_2_ NPs for cell culture experiments

The dye-labelled silica NPs used in this study were synthesized in Lab 1 (Berlin).

#### Synthesis of the dye-label

**Figure 1:**
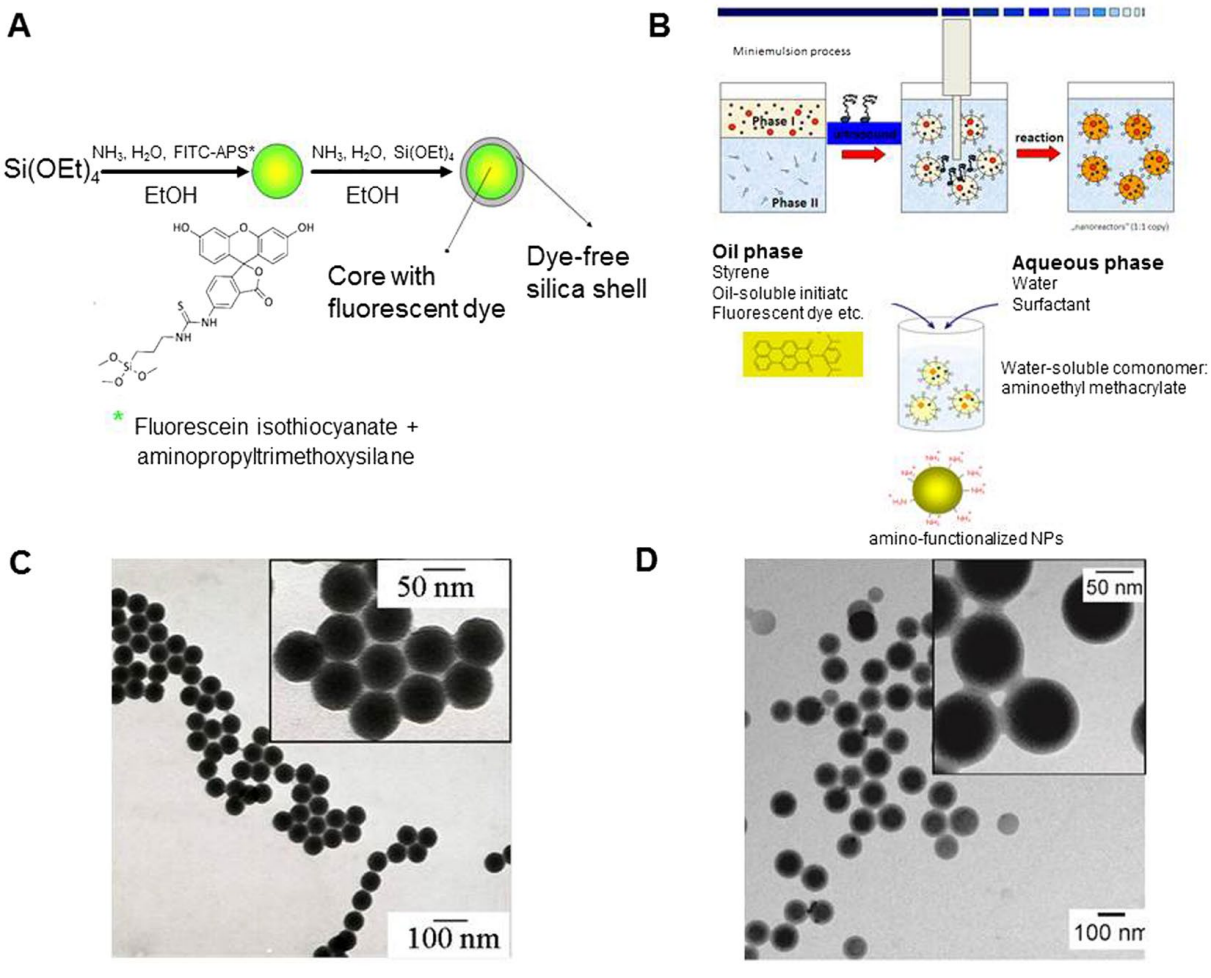
Features of the NPs used in this study. (**A**) Scheme of the synthesis route of the fluorescein isothiocyanate (FITC) labeled SiO_2_ NPs coated with a dye-free SiO_2_ shell (SiO2/FITC NPs). (**B**) Schematic illustration of the synthesis of the polymer NPs, synthesized via miniemulsion synthesis. (**C**) Transmission electron micrographs of the SiO2/FITC and (**D**) transmission electron micrographs of the polymer NPs.

The coupling product of APS and FITC was synthesized under argon by a modification of the synthetic route described by Imhof *et al*.^15^. Dye-labelled SiO2 NP cores covalently labelled with this coupling product were prepared by a modified microemulsion synthesis, as described before^4^ (see reaction scheme in Fig. 1A). The resulting particles were purified three times using repeated sedimentation/redispersion cycles to remove any unbound dye. Subsequently, a SiO_2_ shell was grown on the labeled SiO_2_ cores by a Stöber-like growth process and the resulting particles were again purified by repeated sedimentation/redispersion cycles. The reaction vessels used for reactions yielding SiO_2_ NPs were etched before synthesis with hydrofluoric acid (8 vol.-%) in order to remove insoluble SiO_2_ residues from the glass surface. Subsequently, the vessels were rinsed with ultrapure water. An ultrasonic bath (Bandelin Sonorex, RK 512 H, 860 W) was used for redispersion of sedimented NPs.

#### Transfer of dye labelled NPs from ethanol to DMEM/FCS

The transfer process of the dye labeled SiO_2_ NPs from ethanol into a standard cell culture medium (DMEM)/FCS serum solution was carried out in the dark. Ethanol was removed by centrifugation (533 g, 1 h, T = 20 °C). The nanoparticle sediment was redispersed in ultrapure water using ultrasonification (Bandelin Sonorex, RK 512 H, 860 W) for 10 min in a small round-bottom centrifuge tube (BRAND, 780762, 1.5 mL centrifuge tube) or a large (50 mL) conical-bottom polypropylene standard centrifuge tube. The centrifuge tubes should be so deeply immersed into the ultrasonic bath that the dispersion inside the tubes is completely surrounded by the bath liquid. This procedure is repeated thrice to guarantee that the ethanol content was negligible (<0.5 vol-%) and no ethanol-related cytotoxicity can occur in the subsequent cell exposure experiments.

#### Characterization of silica NPs

SiO_2_ NP formulations were characterized with respect to their size by TEM and DLS in Lab 1 (Berlin) and Lab 4 (Duisburg-Essen). The particles were stored and handled in an argon atmosphere until they were transferred to the cell culture medium and added to cells. TEM images (Fig. 1C) were acquired using SiO_2_ NPs from ethanol dispersion. A NP diameter of 55 ± 2 nm was determined from the quantitative evaluation of these images. Zeta potential and dynamic light scattering measurements were performed using a Delsa Nano C from Beckman Coulter at a fixed scattering angle of 165° revealing a Zeta potential of −52 ± 3 mV in water. The non-filtered NPs were measured in ethanol, ultrapure water, and DMEM/FCS with 10% FCS at 25 °C and 37 °C. The particle concentration of all samples was 0.5 g/L for all dynamic and electrophoretic light scattering measurements. Complementing the cell experiments, all NPs used in this work were additionally incubated at 37 °C for 6, 12, 24, 48 and 72 hours with DMEM (containing 10% fetal calf serum) and then characterized by DLS. In these measurements a self-designed dynamic light scattering (DLS) setup was used in Lab 4^16^. Briefly, this setup includes the 532 nm line of a DPSS-Laser (Polytec; Gem, Nd:YAG) for illumination. Scattered light was detected at a 90° angle by a photomultiplier tube (Perkin und Elmer; MP-943), processed by a hardware-correlator (Correlator Ltd.; flex99r-12) and evaluated using a self-developed LabView routine.

### Synthesis and characterization of polymer NPs

Dye-labeled polymer NPs used in this work were synthesized and functionalized in Lab 2 (Mainz).

#### Synthesis of the dye-label

The fluorescent dye Bodipy-1, a polymerizable derivative of borondipyrromethene, was used for labelling the polymer NPs and was synthesized according to Garcia-Moreno^17^. Bodipy-1, which has the maximum of absorption and emission at 523 and 536 nm, respectively, is part of the polymer and thus no leakage of the dye from the nanoparticle can occur.

#### Synthesis of dye-labelled NPs

Polymeric NPs were synthesized by the free-radical mini-emulsion polymerization process as described before^18, 19^. This process is schematically depicted in Fig. 1B.

#### Characterization of polymer NPs

TEM images of the functionalized polystyrene NPs were acquired after drop casting of the NP dispersion onto a carbon-coated copper grid u (Fig. 1C). A NP diameter of 74 ± 11 nm was determined from the quantitative evaluation of these images. Hydrodynamic particle size and size distribution were determined by DLS using a Nicomp 380 sub-micrometer particle sizer (Nicomp Particle Sizing Systems, U.S.A.) at 90° scattering geometry. Zeta potential measurements were performed in a 1 mM KCl solution at pH 7.2 and 23 °C using the Smoluchowski equation^20^ with a detected value of −49 +/− 9 mVmV. The surface charge density was determined by means of polyelectrolyte titration. Particles were titrated with 10^−3^ M cationic polyelectrolyte polydiallyldimethyl ammonium chloride (PDADMAC). The measurements were carried out with 10 mL of nanoparticle sample in aqueous solution with a solid content of 1 g/L. In addition, the polymer NPs were also incubated at 37 °C for up to 72 h with DMEM (containing 10% fetal calf serum) and then characterized in Lab 4 (Essen).

### HepG2 cell cultures

HepG2 cells, derived from human liver carcinoma (CLS- Cell Lines Service, Germany) were used between passages 20 and 30 and cultured in DMEM:Ham’s F12 with L-glutamin supplemented with 10% FCS (Life Technologies) at 37 °C in a 5% CO_2_ humidified environment. No antibiotics were used. At 85% confluence, cells were harvested after washing with Hank´s BSS using 0.25% Trypsin/0.02% EDTA (3 min, 37 °C) and sub-cultured in 75 cm^2^ flask, slides or 96-well plate with a density of 1.8 * 10^4^ cells/cm^2^. They were allowed to grow for 24 h before nanoparticle exposure. The cells were tested for mycoplasma contamination routinely via PCR. The HepG2 cultures were expanded by one laboratory and distributed to all other ones. Handling and other parameters were standardized among the different partners of the inter-laboratory experiment.

#### Cell viability assays

Cell viability assays were performed under standardized protocols using HepG2 cells in Lab 2, 3 and 5. The tested nanoparticle concentrations ranged from 0 to 500 µg/mL. Cells were treated for the incubation times 6, 12, 24, 48 and 72 h.

#### Cellular dehydrogenase activity and cellular ATP-levels

The metabolic dehydrogenase activity and the relative cellular ATP-levels of HepG2 cells were assessed after exposure to different nanoparticle concentrations in Lab 3 (Jena). Free NPs were removed by washing. The cellular dehydrogenase activity was measured by reduction of MTS tetrazolium salt to the water-soluble formazan product by living cells at an absorbance of 490 nm. To measure cellular ATP-levels the ATPLite assay (PerkinElmer) was carried out according to the manufacturer’s instructions. The determined dehydrogenase activities and cellular ATP-levels were normalized to non-treated control cells.

#### Cellular dehydrogenase activity, LDH release and release of interleukin 6 and 8

Determination of dehydrogenase activity by AlamarBlue reduction, the LDH release and the release of interleukin 6 and 8 of HepG2 cells were measured in Lab 5 (Karlsruhe) as also described previously^21^. After treatment of HepG2 cells with NPs, samples were centrifuged (5 min at 300 g). The supernatants were removed and used for determination of LDH and cytokine content. AlamarBlue reagent, diluted 1:20 (v/v) with HBSS, was added to the cells and incubated for 2 h. Aerosil 200 NPs (hydrophilic, Evonic) were used as controls for cytotoxicity.

### Detection of internalized NPs

Experiments to investigate the intracellular localization of NPs were performed in Lab 2 (Mainz) and Lab 3 (Jena) using confocal laser scanning microscopy (CLSM). For these experiments the HepG2 cells were seeded in ibiTreat µ-slides (IBIDI, Germany) (Lab 2, Mainz) or in glass chamber slides (BD, Germany) (Lab 3, Jena). After 24 h, NPs were added to the medium at a concentration up to 500 µg/mL. Before CLSM imaging cells were washed with PBS. Live cell images were taken in Lab 2 (Mainz) with a commercial setup (LSM SP5 STED Leica Laser Scanning Confocal Microscope, Leica). There, the cell membranes were stained with CellMaskOrange (2.5 mg·mL^−1^, Invitrogen), and the cell nucleus with DraQ5 (2.5·10^−6^ M, Biostatus). In Lab 3 (Jena), for microscopy observations with the LSM 510 Meta (Carl Zeiss MicroImaging GmbH) cell membrane was stained with Wheat Germ Agglutinin Alexa Fluor® 633 conjugate (Invitrogen) and the nucleus with Hoechst 33258 (Applichem) after fixation of cells with 4% formaldehyde. Fluorescent particles were detected at 533–570 nm. For a control experiment to investigate the clustering and adhesion of the NPs to differently coated culture slides, the SiO_2_ NPs were suspended in complete culture medium by pipetting and then they were added to the slides without any cells for supplementary microscopy studies.

### Flow cytometry

Flow cytometry was used for the detection of nanoparticle uptake and cell viability in Lab 2 (Mainz). For these investigations, fluorescent SiO_2_ or polymeric particles were added to the cell culture medium of HepG2 cells. After the selected times of nanoparticle incubation, adherent cells were detached from the culture vessel, washed with buffer (PBS, Gibco, Germany), incubated with 28.6 µg/mL/ 7-aminoactinomycin (7-AAD). Flow cytometry measurements were performed on a CyFlow ML using FlowMax 2.57 software (Partec, Germany). The FL1 channel (excited by a 488 nm laser line, emission filter 527 ± 12 nm) was used to analyze the uptake of NPs by utilization of a forward/sideward scatter plot, thereby excluding cell debris. For 7-AAD, the events in the cell gate were analyzed on a FL1/FL6 dot-plot and three different populations (viable, apoptotic, dead) were determined. To normalize the median fluorescence values the factor of particle fluorescence intensity was determined, according to Hocherl *et al*.^22^.

### Statistical analyses

The results are expressed as means ± standard deviation of several independent experiments as indicated in the legends. The significance of difference between two mean values was assessed by a two-tailed Student’s *t*-test. A *p-value* < 0.05 was considered to be statistically significant.

All data generated or analyzed during this study are included in this published article (and its Supplementary Information files).

## Results

### Physico-chemical nanoparticle characterization

In addition to the thorough NP characterization following the synthesis procedure, we carried out DLS measurements after incubating the NPs at 37 °C for 6, 12, 24, 48, and 72 h with DMEM, containing 10% fetal calf serum (see Table 1 and supporting information).

**Table 1:** Hydrodynamic diameters of the used nanoparticle formulations incubated in complete culture medium, measured via DLS in Lab 4 (Duisburg-Essen) for variable time periods.

DLS measurement	d_hydr_(t = 0 d) [nm]	d_hydr_ (t = 1 d) [nm]	d_hydr_ (t = 2 d) [nm]	d_hydr_ (t = 3 d) [nm]
SiO_2_ NPs	125 ± 6	132 ± 7	130 ± 7	122 ± 6
Polymer NPs	118 ± 6	117 ± 6	118 ± 6	121 ± 6

These results show that all NPs were indeed colloidally stable under the conditions and on the timescales of our experiments. Moreover, determination of hydrodynamic diameter of NPs immediately after transfer in complete culture medium was consistent among the laboratories (Supplementary Table 1).

### Comparison of results from different cell viability assays

The effects of NPs on cell viability of HepG2 cells were investigated in Labs 2, 3 and 5 using either SiO_2_ or polymer NPs, standardized protocols with regard to concentrations, incubation times etc. Furthermore, the same batch of HepG2 cells and cell culture medium including fetal calf serum was used by all participants. The different laboratories showed similarities of results, varying sensitivities among the used cytotoxicological test systems but also several discrepancies, particularly due to the absence of harmonization of very specific technical details: In addition to the conditions during shipping and transfer mentioned above, it turned out that SiO_2_ NPs tend to aggregate on polymer coated slips (ibidi, Germany) compared to regular glass cover slips.

#### Silica NPs

**Figure 2:**
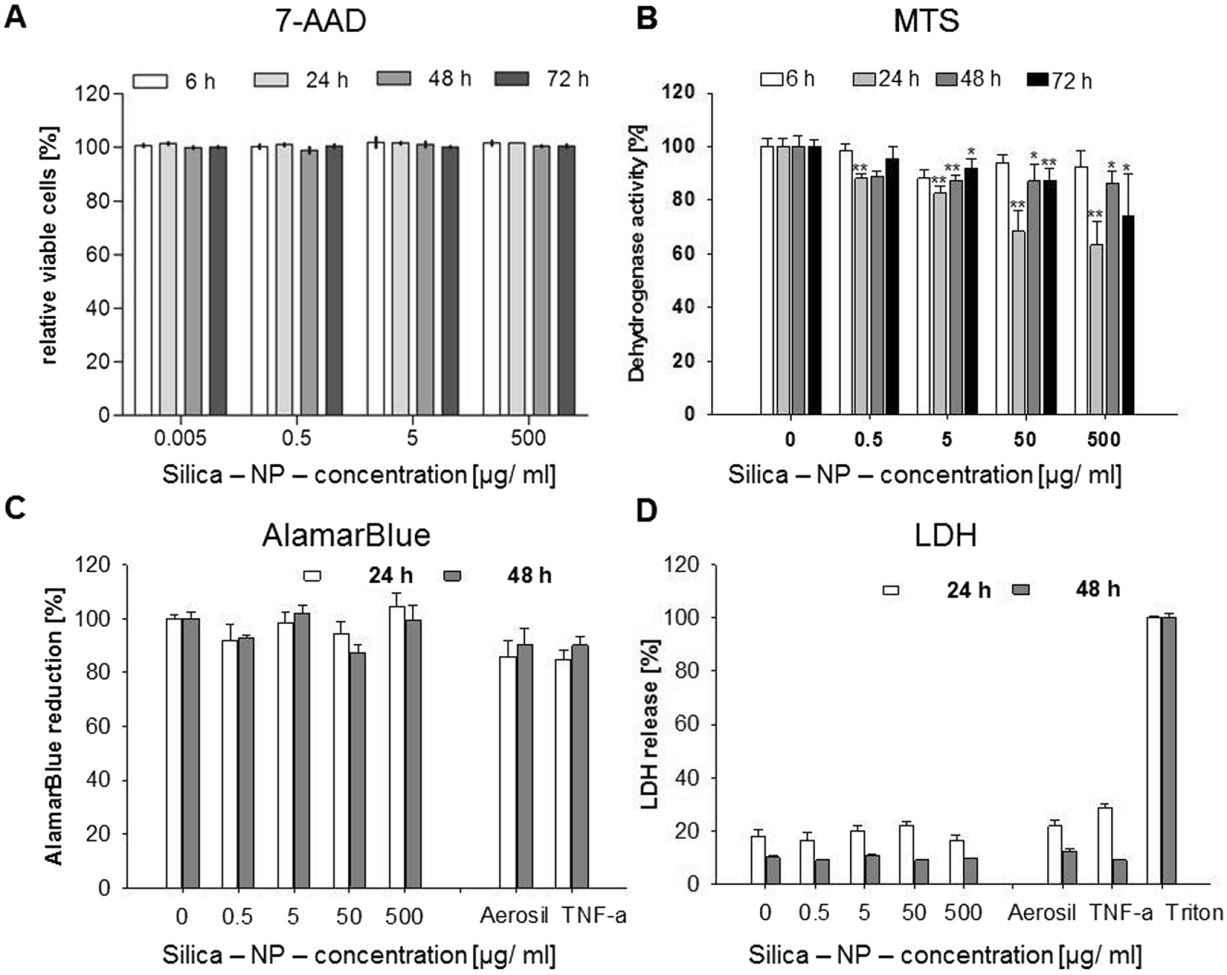
Vitality of HepG2 cells after incubation with SiO_2_-NP measured as determined by different laboratories and read-outs (each partner performed one assay). HepG2 cells were incubated with increasing concentrations of the SiO_2_-NP for different incubation times (up to 72 h). (**A**) Cytotoxicity measurements by flow cytometry (7-AAD staining). (**B**) Effect of SiO_2_-NP on metabolic dehydrogenase activity as detected via MTS-assay. (**C**) Determination of cellular dehydrogenase activity as detected via AlamarBlue assay. (**D**) LDH release after nanoparticle exposure. In (**C**,**D**) 250 µg/mL Aerosil 200-NPs und 20 ng/mL TNF-α were additionally used as controls. All data were normalized to control values (no particle exposure or Triton X-100), which were set as 100%. (**A**) N = 3; B: N = 1, 6 parallels; (**C**,**D**) N = 3, technical duplicates, means and standard deviation (*p < 0.05; **p < 0.01, *** < 0,001 in comparison to untreated controls).

Incubation of HepG2 cells with SiO_2_ NPs lead to only a slight reduction in cell viability in the tested concentration range. The 7-AAD-staining, which demonstrates acute cell death, showed no effect of the SiO_2_ NP, as seen by a 100% vitality at all times after their incubation and for all concentrations (Fig. 2A).

**Figure 3:**
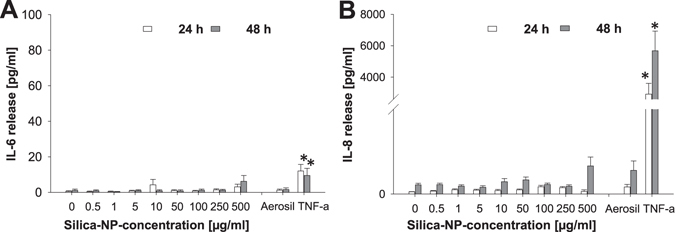
Release of IL-6 (**A**) and IL-8 (**B**) after treatment of HepG2 cells with increasing concentrations of SiO_2_-NPs after 24 and 48 h. 250 μg/mL Aerosil 200 NPs und 20 ng/mL TNF-α were used as positive controls. N = 3, technical duplicates, means and standard deviation (*p < 0.05; **p < 0.01 in comparison to untreated controls).

Via MTS assay, which determines the dehydrogenase activity as a marker of cell metabolism, we observed a slight decrease in the dehydrogenase activity after 24 h at the highest nanoparticle concentrations, but after 48 and 72 h of incubation we observed a recovery in dehydrogenase activity instead (Fig. 2B). This reduction in cell metabolism contrasts with the LDH release, which was not elevated after 24 h and 48 h (Fig. 2D) compared to the non-treated control sample. Also the AlamarBlue test showed at all concentrations and incubation times that cellular dehydrogenase activities were not decreased (Fig. 2C), which indicated a low impact of the SiO_2_ NPs on cell metabolism. The comparison of the different assays indicates that the MTS assay is more sensitive than 7-AAD staining, AlamarBlue reduction and LDH release. Hence, by using only one test, to a certain extent mild effects on cell viability could be missed. Only a slight release of IL-6 and IL-8 occurred after exposure to the highest concentration (500 μg/mL) of SiO_2_ NPs (Fig. 3A and B). This low IL-6 and IL-8 release demonstrated the good biocompatibility of the SiO_2_ NPs, because no inflammatory processes were induced. This result also supports the findings of the vitality assays displayed in Fig. 2.

#### Polymer NPs

**Figure 4:**
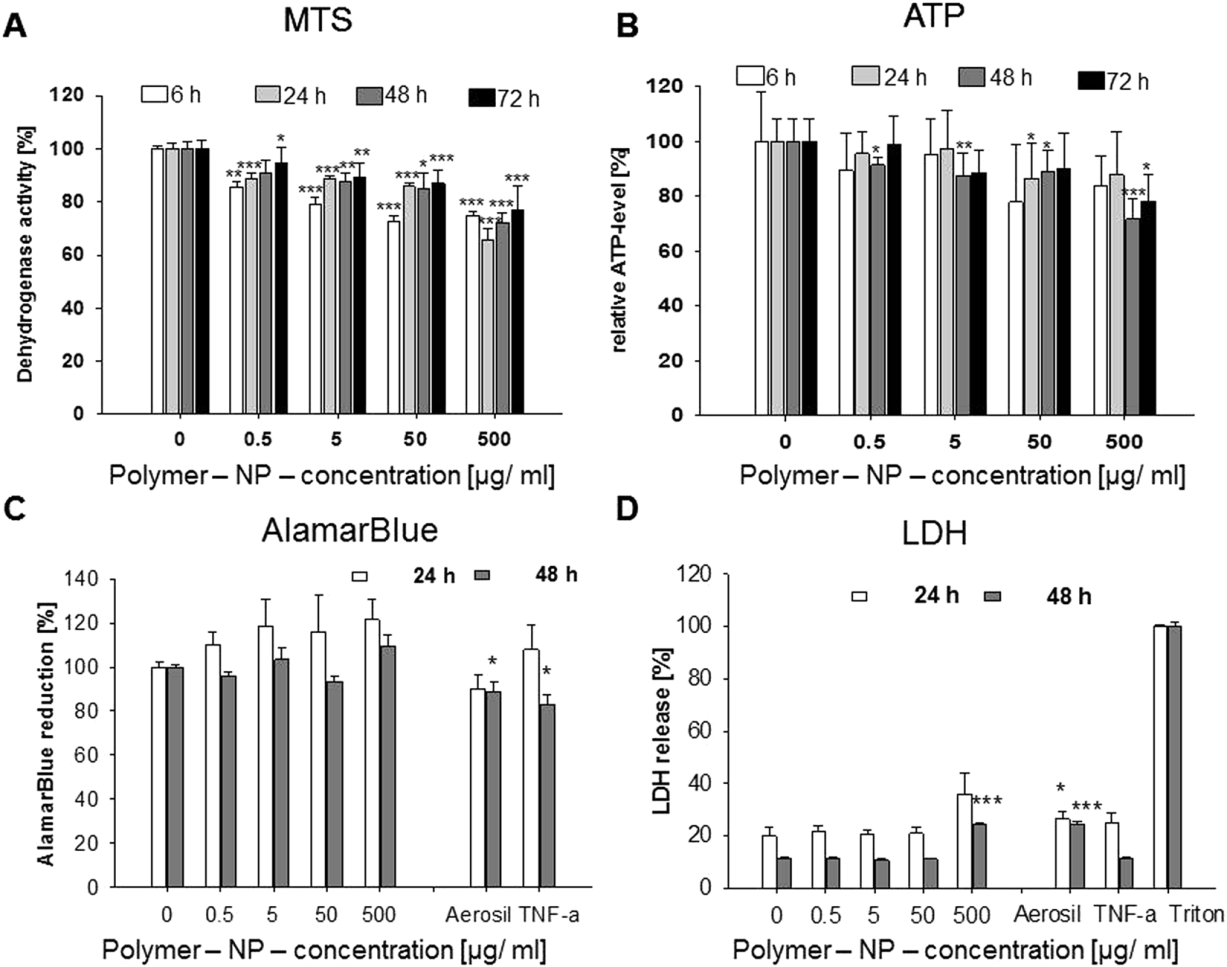
Vitality of HepG2 cells after incubation with polymer-NPs as determined by different laboratories and read-outs. HepG2 were incubated with increasing concentrations of the polymer-NPs for different incubation times (up to 72 h). (**A**) Effect of polymer-NP on metabolic dehydrogenase activity as detected via MTS-assay. (**B**) Relative cellular ATP-levels were detected by ATPLite assay. (**C**) Cellular dehydrogenase activities measured via AlamarBlue assay. (**D**) LDH release after nanoparticle exposure. In (**C**,**D**) 250 µg/mL Aerosil 200-NPs und 20 ng/mL TNF-α were additionally used as controls. All data were normalized to control values (no particle exposure or Triton X-100), which were set as 100%. (**A**,**B**) N = 1 with 6 parallels; (**C**,**D**) N = 3 with 2 parallels, means and standard deviation (*p < 0.05; **p < 0.01, *** < 0,001 in comparison to untreated controls).

Overall and in contrast to SiO_2_ NPs the different test systems showed less consistent results in relation to the polymer NPs. The dehydrogenase activity and the cellular ATP levels decreased consistently in a concentration and time dependent manner (Fig. 4A and B) indicating weak cytotoxic effects at the highest polymer nanoparticle concentration. The LDH release was slightly enhanced at the highest concentration after 24 and 48 h (Fig. 4D). In contrast, the AlamarBlue reduction increased slightly at the highest exposure concentration in comparison to the untreated control (Fig. 4C).

**Figure 5:**
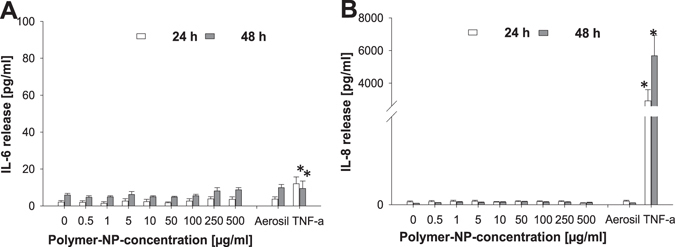
Release of IL-6 (**A**) and IL-8 (**B**) after treatment of HepG2 cells with increasing concentrations of polymer-NPs after 24 and 48 h. 250 μg/mL Aerosil 200-NPs und 20 ng/mL TNFα were used as controls. N = 3, technical duplicates, means and standard deviation (*p < 0.05; **p < 0.01 in comparison to untreated controls).

In conclusion, minor alterations in cellular metabolism are provoked by exposure to polymeric NPs. However, the weak cytotoxic effect monitored by the LDH assay at the highest concentration was not confirmed by 7-AAD staining (Supplementary Figure 1). The IL-6 and IL-8 measurements indicated, similar to the experiments with SiO_2_ NPs, no inflammatory effects of the polymer NPs (Fig. 5). We hypothesize that the cells counter regulate an increase in apoptotic cell death, as supported by MTS, ATP and LDH assays, with an increase of the proliferative activity (AlamarBlue). According to this hypothesis, the results of the various test systems would be consistent and reveal a much more complex image than one test alone could show.

### Uptake of NPs by HepG2 cells

**Figure 6:**
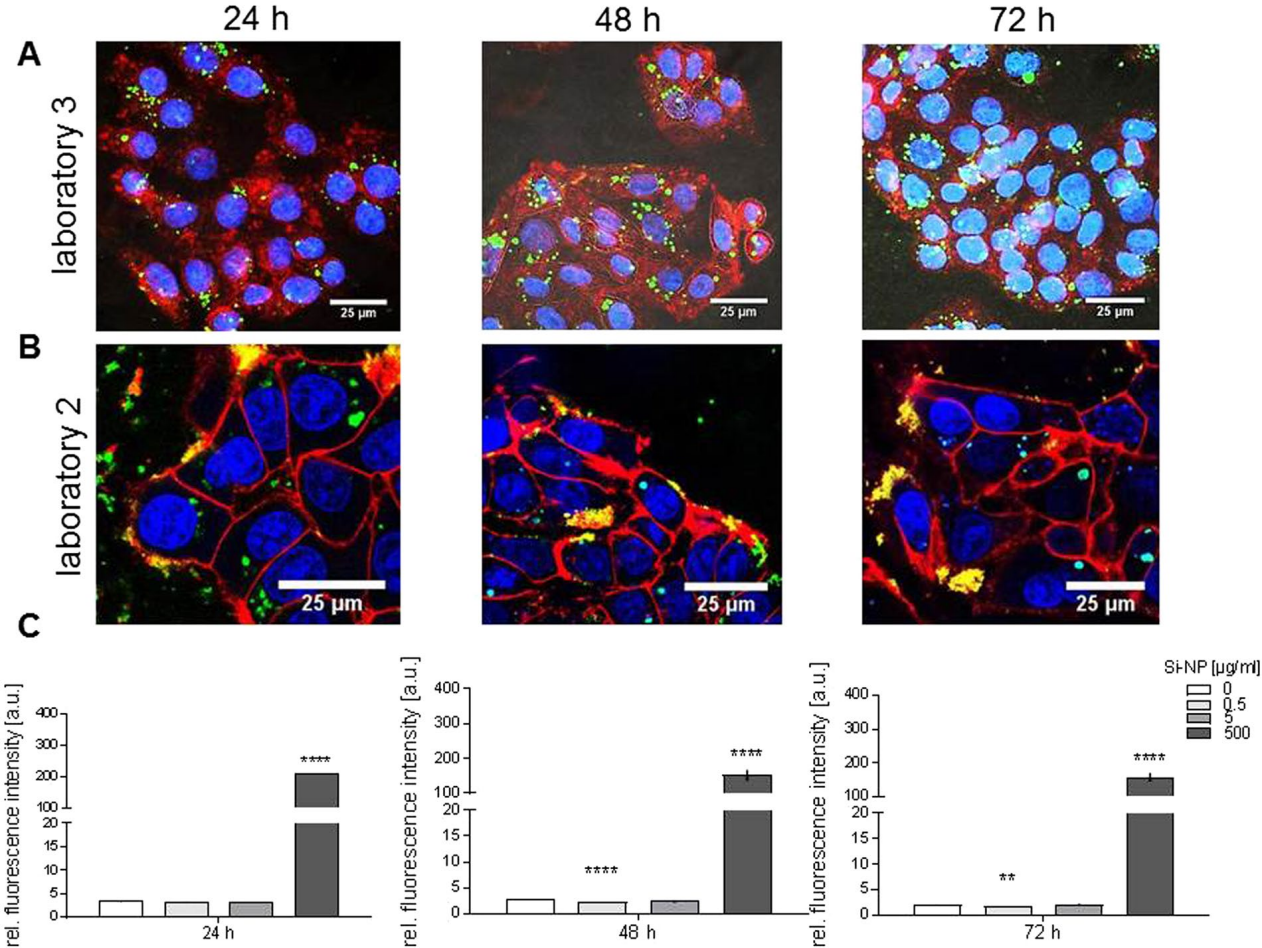
Association of SiO_2_ NPs with cultured HepG2 cells. (**A**) Confocal laser scanning microscopy images of HepG2 after treatment with 500 µg/mL SiO_2_ NPs for 24, 48 and 72 h. (Lab 3). (**B**) Confocal laser scanning microscopy images of HepG2 after treatment with 500 µg/mL SiO_2_NP for 24, 48 and 72 h. (Lab 2). (**C**) Flow cytometry analysis of the uptake after exposure from the experiment conducted by Lab 2. Blue: nucleus, red: cell membrane, green: NPs (**p < 0.01; ****p < 0.0001, compared to untreated controls).

Independent investigations in two different laboratories (Lab 2 and Lab 3) confirmed the uptake of SiO_2_ and polymer NPs into HepG2 cells via CLSM imaging. The SiO_2_ NPs were internalized after 24 h with a perinuclear localization (Fig. 6A and B). However, the CLSM images of Lab 3 indicate an intracellular accumulation of the NPs as evidenced by the green fluorescence signal (Fig. 6A), Lab 2 detected beside large perinuclear aggregates, also a high interaction with the cell surface (Fig. 6B, as probed by the yellow fluorescence caused by colocalization of red (membrane) and green (nanoparticle) fluorescence.

A possible explanation for the discrepant results of the two laboratories could be different gain adjustment of the signal detectors (photomultiplier tubes) as well as different surfaces of the cover slips used for cultivation of cells before analysis by CLSM (a parameter which was not harmonized beforehand). Indeed, SiO_2_ NP were detected as larger agglomerates on polymer (ibidi) coated compared to regular glass cover slips (Fig. 6B, 24 h), which might affect cellular localization and uptake. A control experiment without cells confirmed the finding that SiO_2_ NPs were more agglomerated on the ibidi than the glass surface (Supplementary Figure 2).

**Figure 7:**
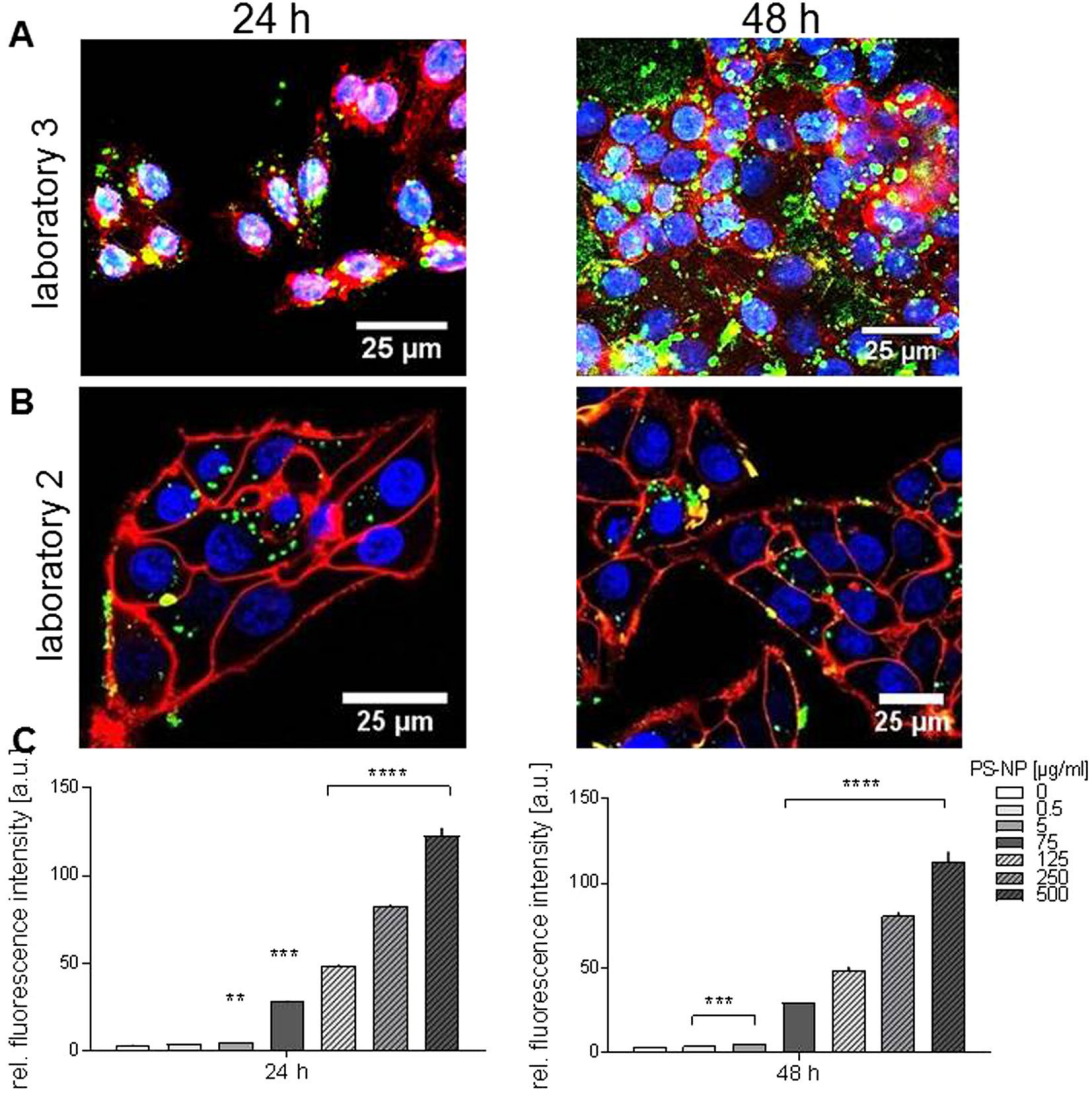
Internalisation of polymer NPs into cultured HepG2 cells. Confocal laser scanning microscopy images of HepG2 after treatment with 500 µg/mL polymer-NP for 24 and 48 h in (**A**) Lab 3 and in (**B**) Lab 2. (**C**) Flow cytometry analysis of the uptake from the experiment conducted by Lab 2. Blue: nucleus, red: cell membrane, green (**p < 0.01; ***p < 0.001; ****p < 0.0001 compared to untreated controls).

The polymer NPs also showed a perinuclear internalization pattern inside of HepG2 cells (Fig. 7). In contrast to the SiO_2_ NPs, the polymer NPs were more adherent to the glass and less to the ibidi surface (Fig. 7A, 48 h). Possibly, this is caused by a repulsion effect between the polymer NPs and the polymer coated ibidi slides. These results show that the outcome of uptake experiments can also depend on the appropriate combination of slide surfaces and nanoparticle chemistry.

Flow cytometry data for the SiO_2_ and polymer NPs further confirmed that the NPs were internalized after 24 and 48 h, if one considers the results of each laboratory independently. Nevertheless, differences in the extent of nanoparticle accumulation in cells between labs 2 and 3 were observed (Figs 6 and 7).

## Discussion

This is the first attempt to cross-validate different assays used for nanoparticle uptake and toxicity studies. All these tests have been employed in the research on nanoparticle toxicity in the literature before, but a direct comparison of their performance in a round robin approach using specified experimental conditions has not been attempted to our knowledge. Here the laboratories involved in the intercomparison study of the DFG Priority Program SPP1313 have agreed on using this array of tests in order to consistently determine their use and differences. Importantly, particular emphasis was devoted to the colloidal stability of the nanoparticle formulations. An accurately defined protocol for the nanoparticle transfer from water into DMEM/FCS led to reproducible colloids for all partner groups within the round robin experiment.

The fact that all NPs were colloidally stable is an important issue for many biological experiments involving NPs^4^. In fact, NP size alterations (in most cases due to agglomeration) will influence its effective density, settling rate and ultimately the effective dose delivered to cells with consideration of a given density and viscosity of the suspension medium. Due to the high ionic strength of cell culture media components, colloids, which are not perfectly stabilized against any interparticular interaction, will form agglomerates with distinctly altered diffusion and sedimentation properties compared to the primary particles. This may lead to a situation where the local NP concentration on the cell surface can be markedly higher than the initial nominal concentration simply by an increased contribution of particle sedimentation. The density of such agglomerates and hence, their sedimentation properties, can be distinctly different from those of the primary particles, i.e. loose agglomerates with a distinct amount of interstitial water sediment slower than compact agglomerates of the same material^23^. Under these conditions, the factual dose rate to the cell membrane is not necessarily a simple function of the initial NP concentration but is significantly affected by NP stability^24^. In case of the SiO_2_ particles (density ≈ 2 g/cm³), sedimentation and diffusion velocities were of the same order of magnitude and the largest agglomerates might have sedimented up to 5–6 times faster than the primary particles. Consequently slight deviations between the expected and the actual NP dose might have occurred. In case of polystyrene particles (density ≈ 1.04 g/cm³) sedimentation is negligible since their density is almost the same like that of water.

When particularly comparing NP cytotoxicity in 2D-cell cultures, one should play particular attention to the utilization of stable NP suspensions, the measuring of the NP size and effective density and the application of sedimentation models to be able to calculate the *actual* dose delivered to cells (for further details see refs 25–27).

In our study, NP dosimetry was not the primary focus, since we aimed at comparing the NP impact on cells among different labs (and not of NP) using identical cell lines and culture medium, identical NP suspensions with verified colloidal stability. Due to the homogeneity of experimental parameters, the NP effective density, fate and transport behavior were expected to be comparable among all the labs involved.

Interestingly, it turned out in the inter-laboratory experiments that small variations of the transfer process for the SiO_2_ NPs can strongly affect the stability and, hence, aggregation state of the NPs in cell culture medium. It took several rounds of careful testing and refining, until NPs from the same batch had the same properties after re-dispersion in the individual labs. Many of the factors that were found to have a distinct impact on the state of the NPs are often not been considered in similar experiments involving single or fewer laboratories. Specifically, it was observed i. e., that the power of the ultrasonic bath, an incomplete immersion of the centrifuge tubes with the NP in the ultrasonic bath, different centrifugal accelerations, and even the geometry of the centrifuge tubes, i.e. the use of small (1–3 mL) conically shaped centrifuge tubes instead of round bottomed or larger conical ones resulted in substantially aggregated samples with hydrodynamic diameters of up to 1000 nm. The impact of sonication parameters such as power output, frequency and sample volume on nanoparticle agglomeration and stability in liquid dispersions has already been shown by other authors^28–30^. The observed drastic effect of the container shape on the sonication processes has not been reported so far. Utmost care should also be taken whenever mailing NPs between laboratories by standard mail, as the conditions during transport are frequently uncontrolled and not reproducible, if the carrier is not well selected. Low (less than <0 °C) and high temperatures (>40 °C) lead to a distinct alteration of the aqueous dispersion of silica nanoparticles. Only the careful elimination of all thermal impact on the samples leads to reliable results by thoroughly standardizing the transport and dispersion procedures. This allowed us to work with high standard samples. In this context it should also be pointed out that such problems can be strongly related to the individual properties of different NPs: the polymer NPs used in this study showed reproducible properties and excellent colloidal stability already in the first round of shipment to the individual groups indicating that they are less prone to be affected by environmental factors such as temperature variations.

From the results presented above we can conclude that the SiO_2_ NPs are not toxic in the tested concentration range. However, there are numerous reports on the potential of SiO_2_ NPs to induce autophagy in liver cells as a result of reactive oxygen species^31^. Yet, in most cases where toxicity of amorphous silica is observed cells are exposed to NPs in the absence of fetal calf serum. Meanwhile it is well known that the presence of serum proteins prevents toxicity of silica NPs in epithelial cells^32, 33^ due to the formation of a protein corona^21^ which most likely also explains the absence of adverse effects in our present study.

Interestingly the polymeric NPs showed in the MTS, ATP and LDH tests a trend towards a moderate toxicity, while the 7-AAD and the Alamar-Blue tests did not indicate this (Fig. 4). This could be due to the different test principles. 7-AAD will determine if the cell is capable of excluding the dye 7-AAD from the inner of each single cell. Therefore it is crucial that the cell is still detectable in the flow cytometer and has not disintegrated into cell debris which is regularly excluded from analysis. Also 7-AAD does include the absolute cell mass as all events on a forward scatter/sideward scatter plot are counted as cells and set as 100%. Of these the cells excluding 7-AAD actively by cell membrane processes are given. 7-AAD can also detect apoptotic and (early) necrotic cells. As stated above very late necrotic events as well as a decrease in proliferation may be missed. MTS and ATP tests in contrary assess the total amount of dehydrogenases or the “total energy level” as ATP. Here doubling or halving cell numbers will result in double or half of the corresponding reading. On the other hand when a cells metabolic capacity is impaired dehydrogenase levels and ATP content may also be decreased. For ATP every energy-consuming event – like even nanoparticle uptake – may decrease the amount of ATP^34^. Therefore these tests are sensitive to several factors unlike the 7-AAD test.

Interestingly, cell viability data between the AlamarBlue test and MTS were slightly different (as shown in Figs 2 and 4) even though both are based on the determination of cellular dehydrogenase activity. Yet, different enzymes might contribute to the metabolism of the dyes in both assays, which could account for the differences observed. Data in the literature about the comparison of different vitality assays are controversial. Hamid *et al*. and Benoit *et al*. reported that Alamarblue is slightly more sensitive with a better reproducibility and a cheaper redox indicator than XTT and MTT^35, 36^. On the other hand, the addition of some compounds to cells like nickel leads to the reduction of Alamarblue so that kinetic experiments may be unreliable unless strictly controlled^37^. Overall, the lack of gross toxicity especially in the presence of serum and efficient uptake of carboxylated polystyrene NPs is in agreement with previous reports^38, 39^. Clearly adsorbed proteins also for polymeric nanoparticles govern uptake^40^ and their intracellular fate^41^.

The differences in the CLSM images between Lab 2 and 3 were not expected and were at first confusing. Although uptake was detected in both labs (Figs 4 and 6), clearly Lab 2 detected more NPs on the cell membrane hereby indicating that NPs had agglomerated before they could have been taken up. After intense discussions we determined that both laboratories had adhered to the standardized parameters, but the choice of the slide chamber used for imaging was not determined beforehand as this was not suspected to have any influence. Interestingly, in the case of silica NPs we could show that nanoparticle agglomeration is higher on the ibidi slide while glass slides did not show this effect. Additionally, the differences of nanoparticle uptake detected by CLSM might be attributed to different sensitivity adjustment of detectors between the laboratories. Furthermore, the comparison of these data with flow cytometry, as a quantitative method, showed that flow cytometry was less sensitive at concentrations below 75 µg/mL due to cellular auto-fluorescence. Hence, using flow cytometry as single method could lead to misleading results.

The importance of interlaboratory evaluation of different toxicity assays in various cell lines has recently been appreciated and several studies approached this issue focusing on multiple aspects relevant for nanotoxicology^42^. Our findings extend th**o**se efforts highlighting not only the relevance of harmonized procedures to prepare NP dispersions but also pinpointing the importance to use several independent toxicity assays and defined cell culture surfaces for proper particle imaging.

## Conclusion

Our round robin test revealed that the outcome of a seemingly simple cytotoxicity assay might be highly dependent upon the respective protocol used in a given laboratory. Even apparently not relevant deviations, such as the choice of cell growth matrix material, have shown to be meaningful. Another aspect refers to the importance of utilization of clearly defined and characterized NP samples. Especially, the procedure to prepare reproducible and stable NP stock dispersions is influenced by multiple factors such as even the geometry of the tube used for resuspension. Moreover, comparative analyses of multiple *in vitro* screening methods are absolutely essential and reliance on a single assay could provide false positive or negative results. In summary, our findings could help in getting more consistent and conclusive data for hazard assessment of nanomaterials in the near future.

## Electronic supplementary material

Supplementary Information
