# Supplementary material for: Validation of weak biological effects by round robin experiments: cytotoxicity/biocompatibility of SiO2 and polymer nanoparticles in HepG2 cells
Source: Sci Rep. 2017 Jun 28;7:4341. doi: 10.1038/s41598-017-02958-9 (PMC5489506; doi:10.1038/s41598-017-02958-9)
Supplement: Supplementary file 1 — Supplementary Information [file 41598_2017_2958_MOESM1_ESM.doc]

**Supplementary Information**

# Validation of weak biological effects by round robin experiments: cytotoxicity/biocompatibility of SiO_2_ and polymer nanoparticles in HepG2 cells

Lisa Landgraf^1^, Daniel Nordmeyer,^2^ Peter Schmiel,^2^ Qi Gao,^2^ Sandra Ritz^3^, Julia Gebauer^4^, Stefan Graß^4^, Silvia Diabaté^6^, Lennart Treuel^4,7^, Christina Graf^2^, Eckart Rühl^2^, Katharina Landfester^3^, Volker Mailänder^3,5^, Carsten Weiss^6^, Reinhard Zellner^4^, and Ingrid Hilger^1*^

^1^ Department of Experimental Radiology, Institute of Diagnostic and Interventional Radiology I, University Hospital Jena, Friedrich-Schiller Universität Jena, Am Klinikum 1, 07747 Jena, Germany

^2^ Physikalische Chemie, Institut für Chemie und Biochemie, Freie Universität Berlin,
Takustr. 3, 14195 Berlin Germany

^3^ Max Planck Institute for Molecular Biology, Ackermannweg 10, 55128 Mainz, Germany

^4^ Institute of Physical Chemistry, University of Duisburg-Essen, 45128 Essen

^5^ Department of Dermatology, University Medicine of the Johannes-Gutenberg University Mainz, Langenbeckstr. 1, 55131 Mainz

^6^ Karlsruhe Institute of Technology, Institute of Toxicology and Genetics, Hermann-von-Helmholtz-Platz 1, 76344 Eggenstein-Leopoldshafen, Germany

^7^ Fraunhofer ICT-IMM, Carl-Zeiss-Str. 18-20, 55129 Mainz, Germany

Corresponding author: Professor Dr. Ingrid Hilger; Institute of Diagnostic and Interventional Radiology I, University Hospital Jena, Am Klinikum 1, 07747 Jena, Germany. Phone: 0049-3641-9325921, Fax: 0049-3641-9325922, e-mail: Ingrid.hilger@med.uni-jena.de

**Supplmentary Table 1:** *Hydrodynamic diameter (d_hydr_) of SiO_2_/FITC nanoparticles after the transfer in DMEM/FCS during the interlaboratory experiments measured at the different locations.*

| Transfer of nanoparticles Location: | d_hydr_ (immediately after transfer) [nm] | d_hydr_ (after 1 days) [nm] | d_hydr_ (after more than 12 days) [nm] |
| --- | --- | --- | --- |
| Lab 2 in Mainz | 120±4 | 110±2 | 107±2 |
| Lab 3 in Jena | 119±5 | - | 114±2 |
| Lab 4 in Essen | 121±4 | - | 119±4 |
| Lab 5 in Karlsruhe | 119±4 | 105±4 | 110±3 |

The nanoparticle dispersions were treated by ultrasound for at least 15 min before each DLS measurement. Immediately after the transfer of SiO_2_ nanoparticles, DLS measurement was carried out locally. Measurements after 1 and more days were done in Lab 1.


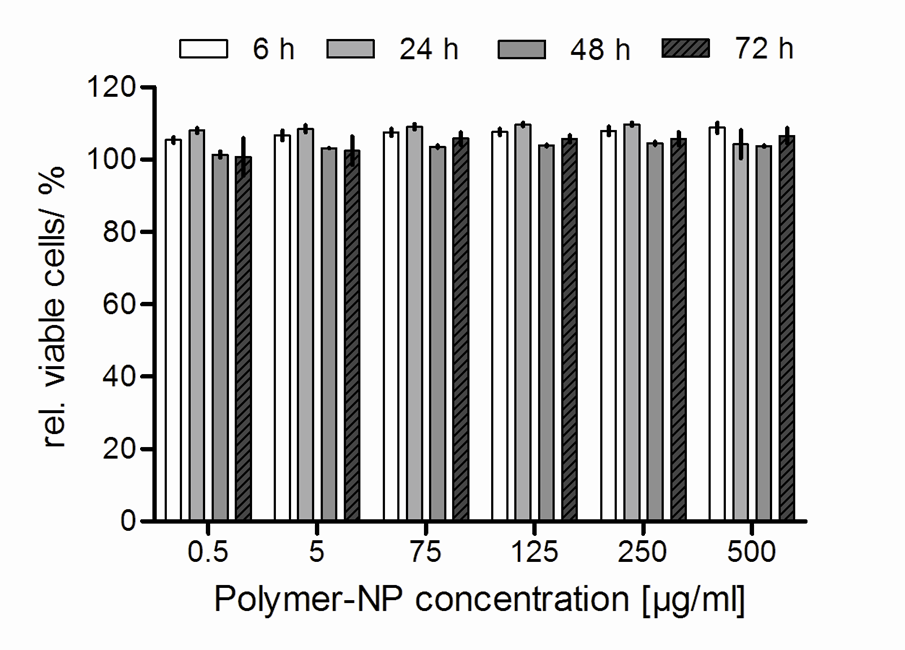


**Supplementary Figure 1. Vitality of HepG2 cells after incubation with polymer-NPs.** HepG2 were incubated with increasing concentrations of the polymer-NPs for different incubation times (up to 72 h). Cytotoxicity measurements were performed by flow cytometry (7-AAD staining).


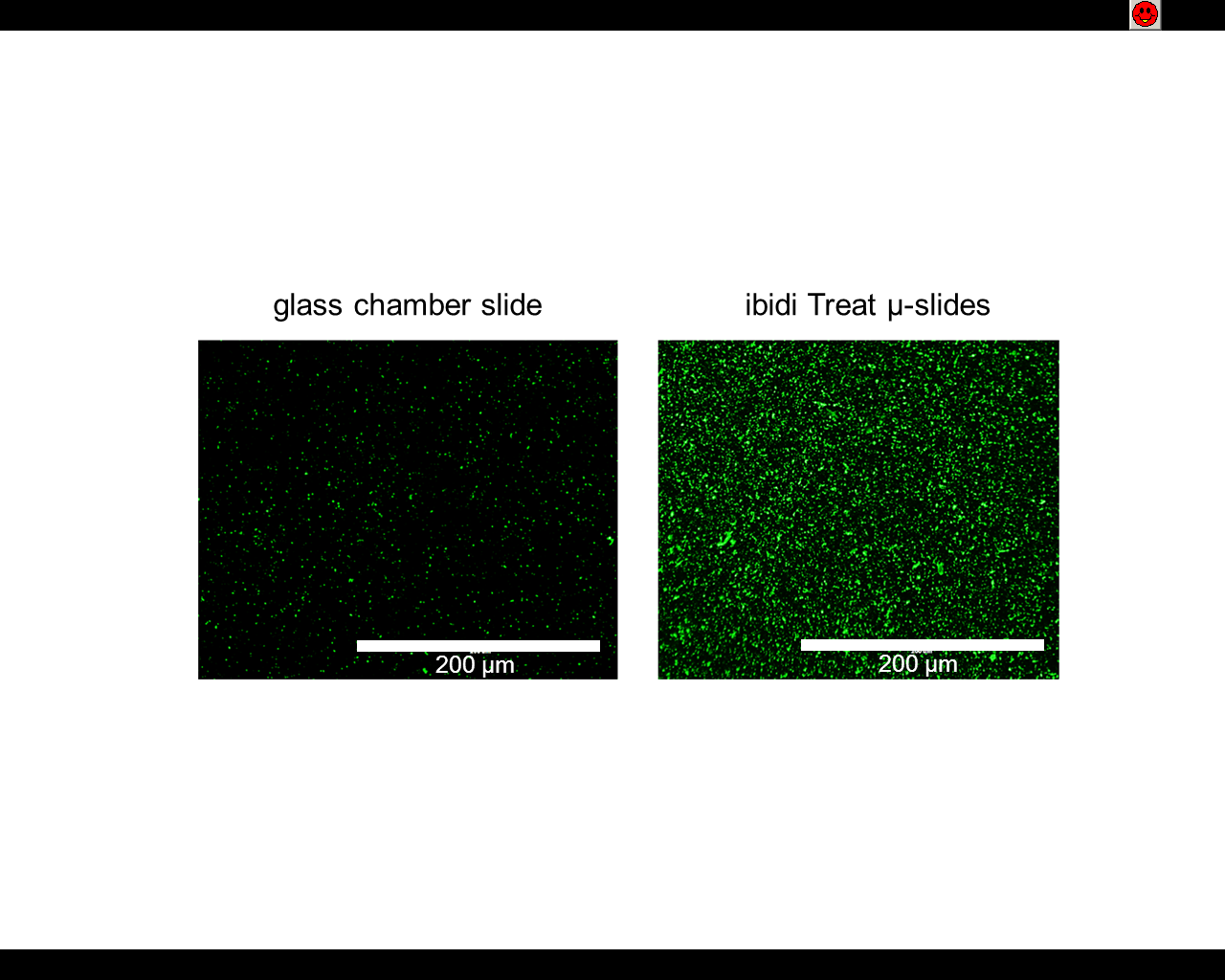


**Supplementary Figure 2**. **Adhesion of SiO_2_ nanoparticles** to the glass chamber slides (left) and to the ibidi slides (right). Nanoparticles are suspended in complete culture medium. Scale bar: 200 µm. Nanoparticles: green
